# *Bubulcus ibis*, *Ciconia ciconia* and *Erinaceus europaeus* from a Wildlife Recovery Center in Portugal as Potential Carriers of Resistant *Escherichia coli*

**DOI:** 10.3390/vetsci12090799

**Published:** 2025-08-23

**Authors:** Sofia Santos, Raquel Abreu, Diana Gomes, Catarina Geraldes, Gonçalo Pereira, Isa Serrano, Eva Cunha, Luís Tavares, María Casero, Manuela Oliveira

**Affiliations:** 1 CIISA—Centre for Interdisciplinary Research in Animal Health, Faculty of Veterinary Medicine, University of Lisbon, 1300-477 Lisbon, Portugal; ss11@edu.ulisboa.pt (S.S.); rmsilva@fmv.ulisboa.pt (R.A.); dgomes@fmv.ulisboa.pt (D.G.); cgeraldes@fmv.ulisboa.pt (C.G.); goncalopereira@fmv.ulisboa.pt (G.P.); iserrano@fmv.ulisboa.pt (I.S.); ltavares@fmv.ulisboa.pt (L.T.); moliveira@fmv.ulisboa.pt (M.O.); 2 Associate Laboratory for Animal and Veterinary Sciences (AL4AnimalS), 1300-477 Lisbon, Portugal; 3 RIAS—Centro de Recuperação e Investigação de Animais Selvagens, 8700-194 Olhão, Portugal; mariavmcasero@gmail.com; 4 cE3c—Centre for Ecology, Evolution and Environmental Changes, CHANGE—Global Change and Sustainability Institute, Faculty of Sciences, University of Lisbon, 1749-016 Lisbon, Portugal

**Keywords:** antimicrobial resistance, virulence factors, ESBL, *Escherichia coli*, wildlife

## Abstract

Antimicrobial resistance (AMR) poses an increasing threat to public health, with wildlife recognized as reservoirs and vectors of resistant bacteria. However, the role of wild species in the ecology of AMR remains insufficiently understood, highlighting the need to investigate resistant bacteria in these animals. This study focused on detecting and characterizing *Escherichia coli* obtained from 43 fecal samples of white storks (*Ciconia ciconia*), cattle egrets (*Bubulcus ibis*), and European hedgehogs (*Erinaceus europaeus*) admitted to a wildlife rehabilitation center in Portugal. Resistance profiles to twelve antibiotics and six virulence factors were characterized phenotypically. ESBL production was also tested. A total of 79 *E. coli* isolates were obtained from 39 out of 43 samples, and 75 were selected for further characterization. All isolates tested negative for ESBL production. Approximately 64% (n = 48/75) of isolates were resistant to at least one antibiotic, and 5.3% (n = 4/75) were multidrug-resistant. Most frequent resistances were to ampicillin (36%, n = 27/75), tetracycline (12%, n = 9/75), and chloramphenicol (8%), while all isolates were susceptible to meropenem, aztreonam, and third-generation cephalosporins. Most isolates (81.3%, n = 61/75) lacked virulence factors. These findings suggest that wildlife may act as a reservoir of resistant strains, emphasizing importance of AMR monitoring and the One Health approach.

## Simple Summary

Antimicrobial resistance (AMR) is a growing problem affecting humans, animals, and the environment. Wild animals can carry bacteria that resist antibiotics, but their role in spreading these bacteria is not fully known. In this study, we studied *Escherichia coli* obtained from the feces of synanthropic species namely white storks, cattle egrets, and European hedgehogs brought to a wildlife center in Portugal. We tested these bacteria against twelve antibiotics, evaluated their ability to produce six different virulence factors, and specifically if they produce extended-spectrum β-lactamases (ESBL). A total of 79 *E. coli* isolates were obtained from 39 out of 43 samples, and 75 were selected for further characterization. We found that 64% (n = 48/75) of the bacteria were resistant to at least one antibiotic, and 5.3% (n = 4/75) resisted at least three classes of antibiotics. Most bacteria (81.3%, n = 61/75) did not produce virulence factors and no ESBL producing *E. coli* was detected. These findings underscore the role of wild animals as potential reservoirs of antibiotic-resistant bacteria, reinforcing their importance in AMR surveillance strategies for public health protection.

## 1. Introduction

Antimicrobial resistance (AMR) is a natural phenomenon that has been exacerbated by the intensive use of antibiotics in human medicine, veterinary practice, and agriculture, representing one of the most significant global public health threats [1]. *Escherichia. coli* stands out in this context due to its ubiquity in the gastrointestinal tract of warm-blooded animals, because of its ability to acquire and disseminate resistance genes, such as beta-lactamases, and as a recognized opportunistic or secondary pathogen [2,3].

Extended-spectrum β-lactamases (ESBL) are enzymes produced by some bacteria, including *E. coli*, that confer resistance to a wide range of β-lactam antibiotics such as penicillins and cephalosporins. The occurrence of ESBL-producing *E. coli* in wildlife is of particular concern, as it indicates environmental dissemination of highly resistant strains that can compromise both human and veterinary antimicrobial therapies [3,4,5]. Monitoring ESBL presence in wild animals contributes to understanding their role in the spread of these critical resistance determinants and their potential impact on public health and ecosystem health [6].

While most AMR studies have focused on clinical settings and food-producing animals, there is a growing recognition of the role of wildlife as a reservoir and potential disseminator of resistant bacteria [7]. Even without direct exposure to antimicrobials, wild animals can acquire resistant strains through contact with environments contaminated by human and agricultural waste [5,7,8]. Their mobility, especially in migratory species, facilitates the intercontinental spread of AMR, connecting ecosystems with varying levels of antimicrobial pressure [2,7]. However, AMR surveillance in wildlife is challenging due to the free-ranging nature of these animals. Wildlife rehabilitation centers, therefore, offer strategic opportunities for monitoring, as they receive individuals from a variety of habitats, often with close contact with urban, agricultural, or industrial areas (environments with a high risk of exposure to resistant microorganisms) [9].

This study aims to investigate the presence and characterize the resistance and virulence profiles of *E. coli* strains isolated from fecal samples of three wild animal species with synanthropic habitats: the white stork (*Ciconia ciconia*), the cattle egret (*Bubulcus ibis*), and the European hedgehog (*Erinaceus europaeus*). These species were chosen because they frequently inhabit human-altered environments, such as agricultural lands, urban areas, and landfills, leading to a high proximity with humans, which may increase their exposure to antimicrobial resistant bacteria.

The white stork is an opportunistic bird that nests in artificial structures and feeds in a wide variety of habitats, including rice fields and landfills [10,11]. The cattle egret, also highly adaptable, forages in agricultural and wetland areas, often in association with large herbivores or agricultural machinery [12,13]. The European hedgehog is a nocturnal, ground-dwelling mammal commonly found in urban and rural environments, with a mainly insectivorous but opportunistic diet [14,15].

By assessing the presence of *E. coli* in these species and characterizing the antibiotic resistance and virulence profiles of the isolates, this study seeks to evaluate their role as potential reservoirs and disseminators of AMR. This research aligns with the One Health approach, contributing to a better understanding of the ecology of AMR in non-clinical settings and highlighting the importance of wildlife in environmental AMR surveillance.

## 2. Materials and Methods

### 2.1. Sampling Location

Samples were collected from March to December 2023 at the Wildlife Rehabilitation and Research Centre—RIAS in Olhão, Faro district, Portugal. All the sampled animals originated from Faro and Beja districts, with municipality of origin recorded.

### 2.2. Sampled Animals

Forty-three samples were collected from wild white storks (n = 21), cattle egret (n = 4), and European hedgehog (n = 18). Animals were admitted to RIAS by private individuals or official authorities (e.g., ICNF, SEPNA or firefighters), with minimal direct human contact expected. Sampling avoided individuals whose welfare or public health could be compromised (e.g., very young—hedgehogs with less than 100 g, milk-fed, and not producing faeces—injured, stressed, or individuals with zoonotic diseases). Data on cause of admission, sampling dates, admission number, species, age, sex and location (municipality of origin) were recorded.

Causes of admission were categorized into six groups: “Trauma,” “Disease,” “Debilitation,” “Orphan/Fallen from nest,” “Illegal captivity,” and “Other.” Animals with physical trauma (fractures, dislocations, wounds, or suspected spinal injuries) were classified under “Trauma.” Animals with lesions compatible with infectious disease, poisoning, inflammation, or infection identified during the physical exam were classified as “Disease.” Individuals showing no visible injuries but appearing dehydrated, emaciated (score 1 or 2/5) [16,17], or lethargic were placed in the “Debilitation” category. Animals rescued from inappropriate locations such as roads or wells, or captured by domestic animals, were classified as “Other”.

Regarding age, animals were categorized as “Nestling”, “Juvenile,” or “Adult.” The distinction between “Nestling” and “Juvenile” was based on size, independence level, type of feeding, and species-specific morphological features, such as feather color and development stage [10,13,14].

### 2.3. Sampling Technique

Fecal samples were collected using sterile swabs with liquid transport medium (Σ-TRANSWAB^®^, MRE Medical Wire, Corsham, Wiltshire, UK). For *B. ibis* and *C. ciconia*, samples were taken before examination or immediately after euthanasia, within a maximum of 5 min, by swabbing the cloaca for ≥3 s. For *E. europaeus*, samples were collected by swabbing the interior of fresh feces, within 24 h post-admission, avoiding contamination. Swabs were refrigerated at 4 °C and transported within 5 h to the Bacteriology Laboratory of the Faculty of Veterinary Medicine, University of Lisbon, Portugal, for further processing.

### 2.4. Isolation and Identification of Escherichia coli

Swabs were pre-enriched in Buffered Peptone Water (VWR, Leuven, Belgium) at 37 ± 2 °C for 18 ± 2 h, then plated on MacConkey agar (VWR, Leuven, Belgium) for selective isolation at 37 °C for 24 h. Up to four colonies with *E. coli*-like morphology (lactose-fermenting colonies appearing pink, often surrounded by a precipitated bile salt halo) were subcultured on Brain Heart Infusion supplemented with 1.4% of bacteriological agar (BHI agar) (VWR, Leuven, Belgium). Then, oxidase production and gram staining were performed on the subcultured isolates. Oxidase-negative, Gram-negative bacilli were confirmed with IMViC test, using Simmons Citrate Agar (Oxoid, Basingstoke, Hempshire, UK), Sulfide Indole Motility Agar (Merck, Darmstadt, Germany), and Voges-Proskauer Broth (Oxoid™, Hempshire, UK). After 24-h incubation at 37 °C, isolates showing indole positivity, methyl red positivity, Voges-Proskauer negativity, citrate negativity, with motility, and absence of hydrogen sulfide production were identified as *E. coli* [18].

For the antimicrobial and virulence evaluation, an *E. coli* collection was established as follows: from fecal samples yielding more than two *E. coli* isolates, only two morphologically distinct colonies (grown on MacConkey agar) were selected for inclusion in the collection.

### 2.5. Antimicrobial Resistance Profile

Antimicrobial resistance profiles were determined via the disk diffusion method according to Clinical and Laboratory Standards Institute (CLSI) guidelines [19,20]. Twelve antibiotics (Oxoid, Hampshire, UK) covering ten antimicrobial classes were tested: aminoglycosides—gentamicin (CN, 10 μg); penicillins—ampicillin (AMP, 10 μg); third-generation cephalosporins—ceftazidime (CAZ, 30 μg) and cefotaxime (CTX, 30 μg); fluoroquinolones—enrofloxacin (ENR, 5 μg) and marbofloxacin (MAR, 5 μg); carbapenems—meropenem (MEM, 10 μg); tetracyclines—tetracycline (TE, 30 μg); β-lactam/β-lactamase inhibitor—amoxicillin/clavulanic acid (AMC, 30 μg); monobactams—aztreonam (ATM, 30 μg); amphenicols—chloramphenicol (C, 30 μg); and sulfonamides—trimethoprim/sulfamethoxazole (SXT, 25 μg).

These antibiotics were selected based on common use in human and veterinary medicine, within a One Health framework, and their relevance to the wildlife rehabilitation setting. Quality control included testing *E. coli* ATCC^®^ 25922™ [20].

Inhibition zone diameters were measured (mm) to classify isolates as susceptible (S), intermediate (I), or resistant (R), according to CLSI breakpoints [19,20]. A 10% replicate analysis was performed on randomly selected isolates to verify reproducibility.

### 2.6. ESBL Phenotype Confirmation

Detection of the ESBL phenotype was performed using a double disk synergy test [21,22]. Single colonies were suspended in 3 mL of 0.85% NaCl, adjusted to 0.5 McFarland standard turbidity, and spread on Müeller-Hinton agar (Oxoid, Hampshire, UK) using sterile swabs. Three antimicrobial disks were sequentially placed on the surface of the agar, while being kept at a distance of 20 mm, as follows: CAZ and CTX were placed in each end, and AMC in the middle. The agar plates were then incubated at 37 °C for 16 to 18 h, after which the isolates were classified as ESBL-producers if the inhibition halo surrounding any of the two cephalosporins showed a clear-cut increase towards the AMC disk. *Klebsiella pneumoniae* CECT 7787 and *E. coli* ATCC^®^ 25922 served as positive and negative controls, respectively. A 10% sample subset was re-tested for consistency.

### 2.7. Virulence Phenotype Profile

Virulence phenotypes were assessed by testing the production of five enzymes linked to bacterial pathogenicity and the ability of biofilm formation. Isolates were pre-cultured on BHI agar at 37 °C for 24 h. A 10% replicate was performed for each test.

Protease activity was evaluated using Skim Milk powder (VWR, Leuven, Belgium) supplemented with bacteriological agar (VWR, Leuven, Belgium). Isolates showing a clear halo or reduced opacity around the colonies were considered positive for protease production. *Pseudomonas aeruginosa* ATCC^®^ 27853™ was used as the positive control and *Staphylococcus aureus* ATCC^®^ 29213™ as the negative control [18].

Deoxyribonuclease (DNase) activity was assessed by inoculating the isolates onto DNase agar plates (Remel, Lenexa, KS, USA) and incubating at 37 °C for 24 h. After incubation, 1 mL of a 1:12 dilution of 12N hydrochloric acid (37%) was added to each plate. After 5 min, isolates presenting a clear halo around bacterial growth were considered positive for DNase production [23]. In this test, *S. aureus* ATCC^®^ 29213™ and *E. coli* ATCC^®^ 25922™ were used as positive and negative controls, respectively [18].

Gelatinase production was detected using test tubes containing Nutrient Gelatin tubes (Oxoid, Hampshire, UK). After incubation at 37 °C for 48 h, the tubes were chilled on ice for 15 to 30 min. Isolates that liquefied the medium completely or partially were considered gelatinase-positive [24]. *P. aeruginosa* Z25.1 was used as the positive control, and *E. coli* ATCC^®^ 25922™ as the negative control [18].

Lecithinase activity was evaluated by inoculating isolates on Tryptic Soy Agar (VWR, Leuven, Belgium) supplemented with 10% egg yolk (VWR, Leuven, Belgium). After incubation at 37 °C for 48 h, isolates that produced a white, opaque, and diffuse precipitate around the bacterial growth were considered positive for lecithinase production. In this test, *P. aeruginosa* ATCC^®^ 27853™ and *E. coli* ATCC^®^ 25922™ were used as positive and negative controls, respectively [18].

Hemolysin production was tested by inoculating the isolates on Columbia agar plates supplemented with 5% sheep blood (bioMérieux, Marcy l’Étoile, France), followed by incubation at 37 °C for 24 h [25]. Isolates producing a clear, complete halo around colonies were classified as β-hemolytic, those with an incomplete greenish or brownish halo as α-hemolytic, and those with no halo as non-hemolytic. *S. aureus* ATCC^®^ 29213™ and *Enterococcus faecalis* ATCC^®^ 29212™ were used as positive and negative controls, respectively [18].

Biofilm production was assessed using BHI agar plates supplemented with 0.08% Congo Red reagent (Sigma, Steinheim, Germany) and 5% sucrose (Sigma, Steinheim, Germany). Plates were incubated at 37 °C for 72 h, and results were recorded at 24, 48, and 72 h. Isolates were considered biofilm-positive if colonies appeared partially or fully blackened with a dry and crystalline texture [18]. *Enterococcus hirae* ATCC^®^ 10541™ was used as the positive control and *E. coli* ATCC^®^ 25922™ as the negative control [18,26].

### 2.8. Statistical Analysis

**Figure 1:**
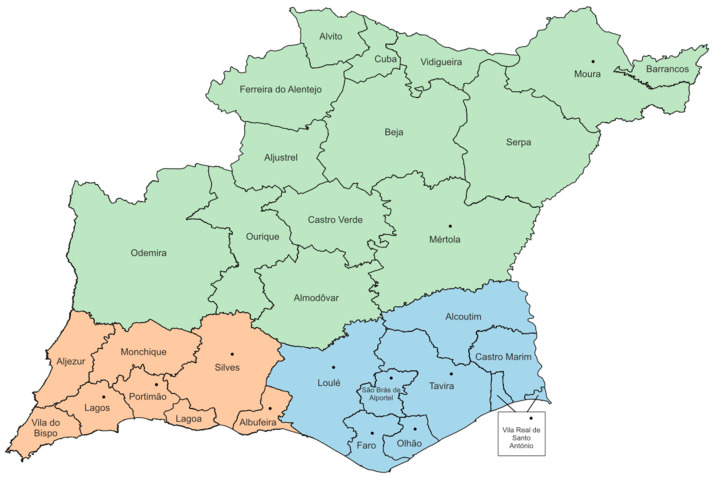
Faro and Beja districts, in Portugal. Municipalities in orange correspond to the Barlavento Algarvio region; municipalities in blue correspond to the Sotavento Algarvio region; municipalities in green correspond to the district of Beja; and municipalities marked with a black dot represent the 12 locations of the animals from which samples were collected (Original map created in QGIS^®^, version 3.40.9).

To address the challenge of having several municipalities/locations (12 total) in the study, municipalities were grouped into three regions to standardize sample distribution (Figure 1):

- Region 1 (Beja): municipalities in Beja District, ecologically distinct from Faro;
- Region 2 (Western—Barlavento Algarvio): municipalities in western Faro District, near Atlantic Ocean;
- Region 3 (Eastern—Sotavento Algarvio): municipalities in eastern Faro District, near Spain.

The Multiple Antimicrobial Resistance Index (MAR Index) and Virulence Index (V. Index) were calculated for each *E. coli* isolate:

MAR Index = (number of resistant antibiotics/total antibiotics tested); V. Index = (number of positive virulence factors/total of virulence factors tested) [18].

Data were compiled in Microsoft^®^ Excel 365 for descriptive statistics, with subsequent analysis in SAS 9.4 (SAS Institute Inc., Cary, NC, USA).

Multivariable logistic regression (PROC LOGISTIC) evaluated associations between independent variables (species, age, sex, cause of admission, location) and isolates’ expression of virulence or resistance determinants; the same model assessed *E. coli* presence relative to animal species.

Since MAR and V. Index values were ordinal, cumulative logistic regression (PROC LOGISTIC) were used to analyze associations with these variables. Models were built with stepwise backward elimination for variables with *p* > 0.157; final models considered *p* ≤ 0.05 significant, and 0.05 < *p* ≤ 0.10 a trend [27].

Multidrug-resistant (MDR) isolates were classified according to the criteria of Magiorakos et al. (2012) as those showing non-susceptibility to at least one agent in three or more antimicrobial categories [28]. For both MAR Index and MDR classification, intermediate results were considered resistant/non-susceptible.

Spearman’s rank correlation (PROC CORR) assessed correlations between MAR and V. Indices, MAR and biofilm production, resistance to different antibiotics (excluding ceftazidime, cefotaxime, meropenem, aztreonam—all uniformly susceptible), and among virulence factors (excluding DNase, gelatinase, lecithinase—all negative). Only correlations with *p* ≤ 0.05 and ρ ≥ 0.40 were considered relevant.

## 3. Results

### 3.1. Sampled Animals

For the study, a total of 43 animals were sampled, distributed among three species: white storks (n = 21; 49%), European hedgehogs (n = 18; 42%), and cattle egrets (n = 4; 9%) (Supplementary File S1).

Regarding sex, the majority of individuals were classified as undetermined sex (n = 31; 72%), including 15 European hedgehogs, 12 white storks, and four cattle egrets. Six males (14%) were identified, all belonging to the species *C. ciconia*, and six females (14%), including three white storks and three European hedgehogs.

Concerning age, 46% of the animals were Nestling (n = 20), 26% Juveniles (n = 11), and 28% Adults (n = 12). Regarding the reasons for admission, the most frequent cause was Orphan/Fallen from nest (n = 11), followed by trauma (n = 10), debilitation (n = 8), Other (n = 7), Disease (n = 5), and Illegal captivity (n = 2). None of the sampled animals were considered to be infected with *E. coli*.

Out of the 43 sampled animals, 17 were released, including 12 hedgehogs and 5 storks.

### 3.2. Identification of the Isolates

A total of 160 isolates exhibiting a macroscopic profile on MacConkey agar compatible with lactose-positive Enterobacteriaceae (pink colonies with a pink precipitate halo corresponding to bile salt precipitation) were obtained from the 43 fecal samples collected. Lactose-fermenting colonies could not be obtained from three of the analyzed samples.

Subsequently, the Indole, Methyl Red, Voges–Proskauer, and Citrate test (IMViC test) was used to confirm *E. coli* identification, from morphologically distinct isolates per sample on MacConkey agar. In total, 109 isolates underwent the IMViC test, and 79 were identified as belonging to the *E. coli* species (Supplementary File S2). Overall, *E. coli* was isolated from 91% (n = 39) of the analyzed samples (n = 43), since one sample did not reveal the presence of *E. coli* among the tested isolates (Table 1).

The four samples from which *E. coli* could not be isolated were excluded from the statistical analysis, with two samples originating from *C. ciconia* and the other two from *E. europaeus*. Statistical analysis revealed no significant relationship between *E. coli* isolation and animal species (*p* > 0.05).

A total of 75 isolates were included in the characterization of virulence and antimicrobial resistance profiles, based on the criterion of selecting two morphologically distinct colonies per sample when multiple isolates were obtained from the same sample.

### 3.3. Characterization of the Isolates’ Antimicrobial Resistance Profile

The resistance profile of 75 out of the 79 identified *E. coli* isolates (approximately two isolates per sample) was evaluated. It was observed that only 27 isolates (36%) were susceptible to all tested antimicrobials, while 64% (n = 48) showed resistance or intermediate resistance to at least one of the 12 compounds analyzed.

**Figure 2:**
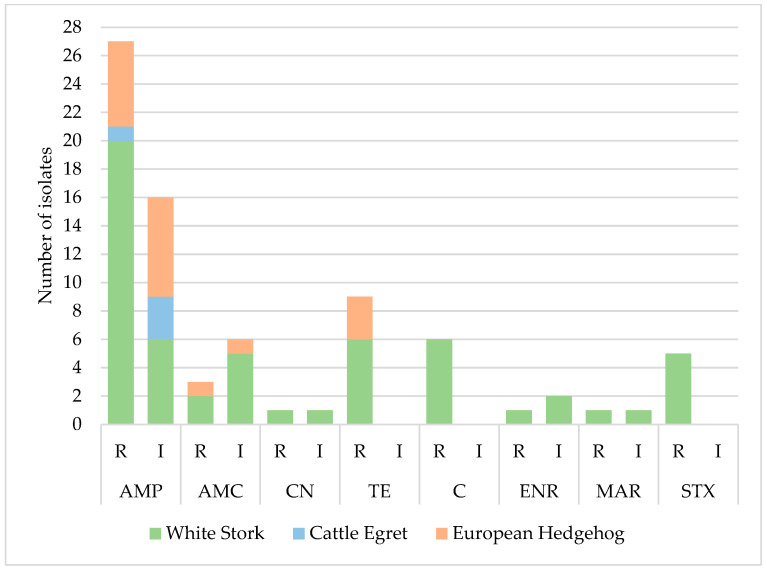
Distribution of antimicrobial resistance profiles by animal species. Resistant (R), Intermediate (I); Ampicillin (AMP), Amoxicillin/Clavulanic Acid (AMC), Gentamicin (CN), Tetracycline (TE), Chloramphenicol (C), Enrofloxacin (ENR), Marbofloxacin (MAR), Trimethoprim/Sulfamethoxazole (STX).

The isolates exhibited the highest levels of resistance to ampicillin (36%, n = 27/75), tetracycline (12%, n = 9/75), and chloramphenicol (8%, n = 6/75). All isolates were susceptible to cephalosporins, carbapenems, and aztreonam (Table 2). Analysis of the antimicrobial resistance profile distribution revealed that isolates from white stork samples showed the highest resistance levels, being the main contributors to the elevated resistance percentage observed in the total isolates (36% resistant to ampicillin, 12% to tetracycline, and 8% to chloramphenicol) (Figure 2). Isolates from cattle egret samples showed resistance only to ampicillin (14%, n = 1/7), and isolates from hedgehogs showed resistance to ampicillin (16.1%, n = 5/31), tetracycline (9.8%, n = 3/31), and amoxicillin/clavulanic acid (3.2%, n = 1/31). No statistically significant differences (*p* > 0.05) were found between resistance to any antibiotic and the variables animal species, sex, age, or location. However, a statistical trend was identified regarding chloramphenicol resistance and sex (*p* = 0.08), with isolates from females being less likely to be resistant to this antibiotic compared to those from males (odds ratio value of 0.111, 95% CI: 0.009–1.337).

Some statistically significant correlations were identified between combined resistance to certain antibiotics. A moderate correlation was observed between chloramphenicol resistance and resistance to enrofloxacin, tetracycline, and trimethoprim/sulfamethoxazole, with correlation coefficients of 0.441, 0.496, and 0.512, respectively. Additionally, a strong correlation was found between resistance to trimethoprim/sulfamethoxazole and enrofloxacin, with a coefficient of 0.764. Finally, a moderate correlation was also noted between resistance to marbofloxacin and gentamicin, with a correlation coefficient of 0.486. According to the definitions proposed by Magiorakos et al. [28], 5% (n = 4) of the 75 isolates were classified as MDR. Among the MDR isolates, it is important to note that two were obtained from animals that were later released. The mean MAR index was 0.09, although 16 isolates showed higher values (MAR index ranged from 0 to 0.50).

No statistically significant differences (*p* > 0.05) were identified in MAR index values regarding the sex, age, or species of the animals, nor the cause of admission or location.

### 3.4. ESBL Phenotype

None of the 75 tested isolates showed evidence of ESBL production, as no increase in the inhibition zone was observed around the cephalosporin disks in the presence of clavulanic acid. As such, no ESBL-producing *E. coli* isolates were detected in this study.

### 3.5. Characterization of the Isolates’ Virulence Phenotype Profile

Regarding the phenotypic expression of virulence factors, 75 of the 79 *E. coli* isolates obtained were evaluated, revealing that most isolates did not exhibit the ability to produce enzymes associated with bacterial virulence (Supplementary File S3). Only 3 isolates (4.0%; n = 75) demonstrated the ability to produce beta-hemolysin, while 8 (10.6%; n = 75) produced protease, and 5 (6.7%; n = 75) showed biofilm formation. In isolates where protease activity was observed, it was consistently weak, evidenced only by a slight reduction in the opacity of the medium surrounding bacterial growth, without the formation of clear halos. Similarly, biofilm production was limited, with colonies only partially blackened. The Virulence Index presented a mean value of 0.04. The maximum observed value was 0.33, corresponding to the expression of two virulence factors (protease and biofilm) in two isolates under study, one originating from a hedgehog and the other from a white stork.

No statistically significant differences were detected in the Virulence Index of the isolates concerning the different factors analyzed, including species, sex, and age of the animals, cause of admission, location, or biofilm production (*p* > 0.05).

Additionally, no association was identified between biofilm production and the other evaluated virulence factors, as well as regarding the MAR index and the multidrug resistance profile of the isolates (*p* > 0.05). Finally, no significant correlation was found between the MAR Index and the Virulence Index (*p* > 0.05).

## 4. Discussion

This study revealed relevant information about antimicrobial resistance and virulence factors in *E. coli* isolates from wildlife species in southern Portugal. Fecal sampling using Amies transport swabs proved to be effective, minimally invasive, and easily to be used in wildlife research [18].

The overall isolation rate of *E. coli* was high (90.5% in white storks, 100% in cattle egrets, and 89% in hedgehogs) consistent with previous studies describing *E. coli* as a common intestinal commensal [29,30,31]. Interestingly, birds showed higher isolation rates than mammals, contrary to the findings of Gopee et al. [29], which may partly reflect the larger number of avian samples included.

Resistance to ampicillin was the most frequently observed (35%, n = 75), followed by tetracycline (12%, n = 9/75) and chloramphenicol (8%, n = 6/75). These results align with previous data in wild animals, where penicillins and tetracyclines often show high resistance levels [32,33]. These patterns are also supported by national and European antibiotic usage data, which place these classes among the most prescribed in both veterinary and human medicine [1,4].

Unexpectedly, resistance to amoxicillin/clavulanic acid was low (4%, n = 3/75) despite its widespread use and inclusion in the WHO’s “access” antibiotic list [34]. Although historical data in Portugal support low resistance to this compound in wildlife [33], more recent studies report increasing resistance, highlighting the need for continued surveillance [6]. This low level may reflect limited recent exposure or lower selective pressure in the sampled environments, potentially influenced by ecological factors such as the habitat, and anthropogenic factors including local antimicrobial usage patterns and waste management practices. However, the potential for horizontal gene transfer remains a concern.

Tetracycline resistance, although relevant in this study (12%, n = 9/75), was lower than previously reported in national and international studies, where levels often exceed 50% [6,35]. Its extensive use in livestock production may explain the variation across ecosystems and reinforces the importance of monitoring livestock antibiotic use [1].

The moderate levels of chloramphenicol resistance observed might be influenced by its ban in food-producing animals since 1994 [32]. Nevertheless, resistant strains are still found, possibly due to the persistence of resistance genes [32]. A resistance rate of 8% in this study is lower than that reported by others in Portugal, such as Sabença et al. [6] (22.7%).

Resistance to trimethoprim/sulfamethoxazole (6.7%, n =5/75) was also low, in line with decreasing usage trends [36]. However, this contrasts with studies showing higher levels in Portuguese wildlife [6], suggesting ecological variability or differences in selective pressure between sites.

Fluoroquinolone resistance (5.3%, n = 4/75) was also lower than expected, considering their high usage in livestock in Portugal [37]. Although these compounds are classified as critically important, resistance to these compounds remains relatively contained in wildlife [18].

Resistance to gentamicin was very low (1.3%, n = 1/75), in agreement with previous findings in Portuguese wildlife [33]. However, Sabença et al. [6] reported higher levels (16.4%), reinforcing the idea that AMR profiles can vary significantly between regions and studies.

No resistance was detected to third-generation cephalosporins, aztreonam, or carbapenems. This is an encouraging result, particularly considering the critical importance of these β-lactam antibiotics in human medicine, with aztreonam and carbapenems being reserved as last-resort options [6,34]. Although ESBL-producing *E. coli* have been reported in Iberian wildlife [30], their absence in this study suggests limited dissemination in the sampled ecosystem. Still, the potential for sudden emergence due to mobile genetic elements warrants continued monitoring [3].

Notably, the highest resistance levels were observed in white stork isolates (75.6%, n = 31/41), with ampicillin (48.8%, n = 20/41) being the most common. These results are consistent with studies from Spain reporting high resistance in this species, attributed to their opportunistic feeding behavior, often associated with landfills and urban areas [31,38]. This supports the hypothesis that feeding ecology and exposure to anthropogenic sources directly influence AMR carriage in wildlife. Future studies should investigate the potential sources of AMR in these animals by analyzing both animal samples and key environmental sites, such as landfills.

Cattle egrets and hedgehogs showed lower AMR levels. However, resistance to ampicillin (19.3%, n = 6/31) and tetracycline (9.7%, n = 3/31) in hedgehogs confirms previous findings suggesting that these mammals may serve as environmental reservoirs of resistant strains [9,39]. The lower diversity of resistance in hedgehogs may be due to their more restricted habitat and reduced exposure to human-related sources, unlike birds, which have greater mobility and contact with anthropized areas.

Only 5% (n = 4/75) of isolates met the definition of multidrug resistance, all from white storks. Although this percentage is lower than reported in other Portuguese studies [6,33], it supports the notion of greater exposure of this species to human or agricultural sources of resistance. Two MDR isolates came from individuals that were later released into the wild, raising concerns about the role of wildlife rehabilitation centers in the dissemination of resistant strains. This highlights an urgent need to monitor the microbial profiles of animals prior to release.

Although no genotypic analysis was performed, the observed correlations between resistance profiles may suggest the presence of mobile genetic elements, such as plasmids or integrons, known to carry multiple resistance genes [3]. Performing molecular analyses in future studies would allow for more precise identification of these mechanisms.

A statistical trend was also observed between the sex of the animals and resistance to chloramphenicol. However, this association may be limited by the small sample size and the unequal sex distribution, justifying further studies with stratified and larger sampling.

Regarding the presence of ESBL-producing *E. coli* in Portuguese wildlife, the available data consistently indicate a very low prevalence [6,21]. In the present study, none of the 75 isolates tested positive for ESBL production, supporting this trend. Similarly, in a previous study, only two wild animals, a red fox (*Vulpes vulpes*) and a griffon vulture (*Gyps fulvus*), were found to carry ESBL-producing *E. coli*, representing just 0.93% of the tested population [6]. It is worth noting that those isolates were recovered using non-selective culture media, which may have led to an underestimation of the actual prevalence. Another Portuguese study also detected ESBL production in only 6% (n = 2) of the isolates, as determined by the modified double-disk synergy test [21]. Taken together, these results suggest that ESBL genes are not yet widespread among Portuguese wildlife. This is a positive finding from both a public health and environmental perspective, although ongoing surveillance remains crucial to monitor future trends.

Regarding virulence, most isolates did not express phenotypic virulence factors. Protease activity was detected in 10.6% (n = 8/75), biofilm formation in 6.7% (n = 5/75) and beta-hemolysis in 4% (n = 3/75) of the isolates. These values are lower than those reported in studies conducted in Costa Rica and Portugal [18,21], possibly reflecting differences in host species or ecological conditions.

The absence of a significant correlation between biofilm production and AMR in this study is consistent with some studies [21], although others have suggested a positive association [40]. Similarly, the lack of a relationship between the V. Index and MAR index supports previous results [21], although Fernandes et al. [18] reported a negative correlation.

The combined assessment of virulence and MAR indices classified most isolates as low-risk [22,41]. However, the detection of MDR strains, especially in migratory species such as the white stork, underscores the need for molecular typing and monitoring prior to release. These results highlight the importance of tailored surveillance strategies, including conventional and/or molecular bacterial detection techniques, as appropriate tools to support biosecurity in wildlife rehabilitation and release programs, thereby helping to minimize the risk of disseminating resistant bacteria.

In this context, the One Health approach becomes essential to understanding the circulation of AMR between wildlife, humans, and the environment. The presence of resistant strains in rehabilitated and released animals may pose a risk of disseminating potentially zoonotic agents. Therefore, future studies should include phylogenetic and genomic analyses, as well as longitudinal monitoring before, during, and after the rehabilitation process.

More broadly, these findings contribute to growing evidence that wildlife, particularly species with urban associations, may play an underrecognized role in the environmental circulation of AMR. Monitoring resistance in such species can serve as an early warning system for anthropogenic impacts on microbial ecology, reinforcing the need for cross-sectoral collaboration under the One Health framework.

This study has some limitations that should be acknowledged. First, only phenotypic tests were used, so future research could incorporate molecular approaches to confirm phenotypic findings, as well as evaluate clonal relationships among MDR strains. Second, the sample size, especially for cattle egrets, was small. Third, the absence of selective media may have led to an underestimation of ESBL-producing isolates. In addition to the use of molecular techniques, future studies could aim to increase the number of samples and include environmental samples (such as soil and water from animal collection sites) to provide additional ecological context.

## 5. Conclusions

This study improves our understanding of antimicrobial resistance in *E. coli* from wildlife in southern Portugal. While overall resistance levels were moderate, the presence of MDR strains in white storks suggests possible anthropogenic influence. Notably, no resistance to critically important antibiotics was found, and all isolates tested negative for ESBL production, indicating limited dissemination of these resistance genes in local wildlife.

The low virulence potential observed, along with specific resistance patterns, highlights the need for continued surveillance, particularly in species with close contact with human-altered environments. These findings support the importance of a One Health approach by demonstrating the interconnected risks to animal, human, and environmental health. They reinforce the need for responsible antibiotic use and integrated monitoring to mitigate the spread of antimicrobial resistance and its potential public health impact.

## Figures and Tables

**Table 1:** Number of *E. coli* isolates by animal species.

Scientific Name	Common Name	No. of *E. coli* Isolates [n/N (%)]	Frequency of *E. coli*-Positive Samples [n/N (%)]
*Ciconia ciconia*	White Stork	41/79 (51.9)	19/21 (90.5)
*Erinaceus europaeus*	European Hedgehog	31/79 (39.2)	16/18 (89)
*Bubulcus ibis*	Cattle Egret	7/79 (8.9)	4/4 (100)

**Table 2:** Antimicrobial susceptibility of the *E. coli* isolates under study (n = 75).

Antimicrobial Class	Antimicrobial Compound (Dosage)	*E. coli* Isolates [n/N (%)] ^a^		
		S ^b^	I ^c^	R ^d^
**Penicillins**	Ampicillin (10 µg)	32/75 (42.7)	16/75 (21.3)	27/75 (36.0)
**β-lactam with β-lactamase inhibitor**	Amoxicillin/Clavulanic Acid (30 µg)	66/75 (88.0)	6/75 (8.0)	3/75 (4.0)
**Monobactamics**	Aztreonam (30 µg)	75/75 (100)	0	0
**Cephalosporins**	Cefotaxime (30 µg)	75/75 (100)	0	0
	Ceftazidime (30 µg)	75/75 (100)	0	0
**Carbapenems**	Meropenem (10 µg)	75/75 (100)	0	0
**Aminoglycosides**	Gentamicin (10 µg)	73/75 (97.3)	1/75 (1.3)	1/75 (1.3)
**Tetracyclines**	Tetracycline (30 µg)	66/75 (88.0)	0	9/75 (12.0)
**Phenicols**	Chloramphenicol (30 µg)	69/75 (92.0)	0	6/75 (8.0)
**Fluoroquinolones**	Enrofloxacin (5 µg)	72/75 (96.0)	2/75 (2.7)	1/75 (1.3)
	Marbofloxacin (5 µg)	73/75 (97.3)	1/75 (1.3)	1/75 (1.3)
**Sulphonamides**	Trimethoprim/Sulfamethoxazole (25 µg)	70/75 (93.3)	0	5/75 (6.7)

Legend: ^a^ n (%), number and percentage of isolates; ^b^ S, Susceptible; ^c^ I, Intermediate; ^d^ R, Resistant.

## Data Availability

The data presented in this study are available in the article and Supplementary Materials. More details can be provided upon reasonable request to the correspondence contacts.

## Supplementary Materials

The following supporting information can be downloaded at: https://www.mdpi.com/article/10.3390/vetsci12090799/s1, Supplementary File S1: Information of the sampled animals; Supplementary File S2: Results of IMViC test of the isolates obtained from the sampled animals; Supplementary File S3: Phenotypic resistance and virulence profiles of 75 selected *E. coli* isolates obtained from the sampled animals.

## Abbreviations

The following abbreviations are used in this manuscript:

AMC	Amoxicillin/clavulanic acid
AMP	Ampicillin
AMR	Antimicrobial resistance
ATM	Aztreonam
BHI agar	Brain Heart Infusion supplemented with 1,4% of bacteriological agar
C	Cloramphenicol
CAZ	Ceftazidime
CLSI	Clinical and Laboratory Standards Institute
CN	Gentamicin
CTX	Cefotaxime
ENR	Enrofloxacin
ESBL	Extended-spectrum β-lactamase
I	Intermediate
IMViC test	Indole, Methyl Red, Voges-Proskauer, Citrate test
MAR	Marbofloxacin
MAR Index	Multiple Antimicrobial Resistance Index
MDR	Multidrug Resistance
MEM	Meropnem
R	Resistant
RIAS	Centro de Recuperação e Investigação de Animais Selvagens
S	Susceptible
STX	Trimethoprim/Sulfametoxazole
TE	Tetraciclin
V. Index	Virulence Index
