# Supplementary material for: Bubulcus ibis, Ciconia ciconia and Erinaceus europaeus from a Wildlife Recovery Center in Portugal as Potential Carriers of Resistant Escherichia coli
Source: Vet Sci. 2025 Aug 23;12(9):799. doi: 10.3390/vetsci12090799 (PMC12474354; doi:10.3390/vetsci12090799)
Supplement: Supplementary file 1 [file vetsci-12-00799-s001.zip › Supplementary File S1.pdf]

Supplementary file S1. Information of the sampled animals

Sample (Nº)	Sample Collection Date	Species	Common Name	Sex	Age	Municipality of Origin	Region	Cause of Admission	Clinical Outcome
**1**	28/06/2023	*Ciconia ciconia*	White Stork	I	Nestling	Olhão	SA	Orphan/Fallen from nest	Released
**2**	25/07/2023	*Ciconia ciconia*	White Stork	I	Juvenile	Portimão	BA	Debilitation	Euthanized
**3**	24/07/2023	*Bubulcus ibis*	Cattle Egret	I	Juvenile	Loulé	SA	Trauma	Euthanized
**4**	16/07/2023	*Ciconia ciconia*	White Stork	M	Nestling	Mértola	A	Debilitation	Died
**5**	17/03/2023	*Erinaceus europaeus*	European Hedgehog	I	Nestling	Portimão	BA	Orphan/Fallen from nest	Released
**6**	21/07/2023	*Ciconia ciconia*	White Stork	M	Juvenil	Portimão	BA	Trauma	Died
**7**	10/07/2023	*Ciconia ciconia*	White Stork	F	Juvenil	Silves	BA	Disease	Died
**8**	28/06/2023	*Ciconia ciconia*	White Stork	I	Nestling	Moura	A	Trauma	Died
**9**	25/06/2023	*Erinaceus europaeus*	European Hedgehog	I	Nestling	Albufeira	BA	Trauma	Died
**10**	17/03/2023	*Erinaceus europaeus*	European Hedgehog	I	Nestling	Portimão	BA	Orphan/Fallen from nest	Released

Sample (Nº)	Sample Collection Date	Species	Common Name	Sex	Age	Municipality of Origin	Region	Cause of Admission	Clinical Outcome
**11**	26/07/2023	*Ciconia ciconia*	White Stork	I	Nestling	Silves	BA	Disease	Euthanized
**12**	09/07/2023	*Erinaceus europaeus*	European Hedgehog	I	Adult	Portimão	BA	Other	Released
**13**	07/07/2023	*Ciconia ciconia*	White Stork	M	Nestling	Portimão	BA	Trauma	Died
**14**	25/06/2023	*Erinaceus europaeus*	European Hedgehog	F	Adult	Albufeira	BA	Illegal Captivity	Died
**15**	30/06/2023	*Bubulcus ibis*	Cattle Egret	I	Juvenile	Silves	BA	Debilitation	Died
**16**	07/07/2023	*Ciconia ciconia*	White Stork	M	Juvenile	Faro	SA	Debilitation	Euthanized
**17**	29/06/2023	*Ciconia ciconia*	White Stork	I	Nestling	Portimão	BA	Trauma	Released
**18**	24/07/2023	*Ciconia ciconia*	White Stork	I	Juvenile	Portimão	BA	Disease	Released
**19**	21/07/2023	*Bubulcus ibis*	Cattle Egret	I	Nestling	Tavira	SA	Debilitation	Died
**20**	26/06/2023	*Ciconia ciconia*	White Stork	I	Nestling	Olhão	SA	Orphan/Fallen from nest	Released
**21**	06/07/2023	*Ciconia ciconia*	White Stork	I	Nestling	Faro	SA	Orphan/Fallen from nest	Released

Sample (Nº)	Sample Collection Date	Species	Common Name	Sex	Age	Municipality of Origin	Region	Cause of Admission	Clinical Outcome
**22**	19/07/2023	*Ciconia ciconia*	White Stork	M	Juvenile	Silves	BA	Disease	Euthanized
**23**	17/07/2023	*Ciconia ciconia*	White Stork	F	Juvenile	Lagos	BA	Disease	Died
**24**	23/05/2023	*Bubulcus ibis*	Cattle Egret	I	Adult	Loulé	SA	Trauma	Euthanized
**25**	15/03/2023	*Erinaceus europaeus*	European Hedgehog	F	Nestling	Portimão	BA	Orphan/Fallen from nest	Died
**26**	28/06/2023	*Ciconia ciconia*	White Stork	I	Nestling	Silves	BA	Orphan/Fallen from nest	Released
**27**	23/06/2023	*Ciconia ciconia*	White Stork	I	Juvenile	Silves	BA	Trauma	Euthanized
**28**	24/05/2023	*Ciconia ciconia*	White Stork	I	Nestling	Tavira	SA	Orphan/Fallen from nest	Died
**29**	23/06/2023	*Ciconia ciconia*	White Stork	M	Nestling	Portimão	BA	Debilitation	Died
**30**	28/06/2023	*Ciconia ciconia*	White Stork	F	Nestling	Portimão	BA	Orphan/Fallen from nest	Died
**31**	21/09/2023	*Erinaceus europaeus*	European Hedgehog	I	Adult	Olhão	SA	Illegal Captivity	Released
**32**	19/11/2023	*Erinaceus europaeus*	European Hedgehog	I	Juvenile	Albufeira	BA	Debilitation	Died

Sample (Nº)	Sample Collection Date	Species	Common Name	Sex	Age	Municipality of Origin	Region	Cause of Admission	Clinical Outcome
**33**	17/09/2023	*Erinaceus europaeus*	European Hedgehog	I	Adult	Olhão	SA	Other	Released
**34**	04/09/2023	*Erinaceus europaeus*	European Hedgehog	I	Nestling	Vila Real de Santo António	SA	Orphan/Fallen from nest	Released
**35**	04/12/2023	*Erinaceus europaeus*	European Hedgehog	F	Adult	Albufeira	BA	Other	Released
**36**	11/09/2023	*Ciconia ciconia*	White Stork	I	Adult	Portimão	BA	Trauma	Euthanized
**37**	04/09/2023	*Erinaceus europaeus*	European Hedgehog	I	Adult	Faro	SA	Other	Released
**38**	21/11/2023	*Erinaceus europaeus*	European Hedgehog	I	Nestling	São Brás de Alportel	SA	Debilitation	Died
**39**	22/11/2023	*Erinaceus europaeus*	European Hedgehog	I	Adult	Vila Real de Santo António	SA	Other	Released
**40**	12/12/2023	*Erinaceus europaeus*	European Hedgehog	I	Adult	Loulé	SA	Other	Released
**41**	07/11/2023	*Erinaceus europaeus*	European Hedgehog	I	Nestling	Loulé	SA	Orphan/Fallen from nest	Released
**42**	12/12/2023	*Erinaceus europaeus*	European Hedgehog	I	Adult	Faro	SA	Other	Released
**43**	20/12/2023	*Erinaceus europaeus*	European Hedgehog	I	Adult	Silves	BA	Trauma	Died

Legend: Indeterminate (I); Male (M); Female (F); Sotavendo Algarvio (SA); Barlavento Algarvio (BA); Alentejo (A)
