# Supplementary material for: Bubulcus ibis, Ciconia ciconia and Erinaceus europaeus from a Wildlife Recovery Center in Portugal as Potential Carriers of Resistant Escherichia coli
Source: Vet Sci. 2025 Aug 23;12(9):799. doi: 10.3390/vetsci12090799 (PMC12474354; doi:10.3390/vetsci12090799)
Supplement: Supplementary file 1 [file vetsci-12-00799-s001.zip › Supplementary File S2.pdf]

Supplementary file S2. Results of IMViC test of the isolates obtained from the sampled

Sample (Nº)	Isolate ID	IMViC Test					
		**Glucose**	**Acetoin**	**Mobility**	**Indole**	**H_2S**	**Citrate**
**1**	1mc1	+	-	+	-	+	-
	1mc2	+	-	+	NP	+	+
	1mcII2	+	-	+	+	-	-
	1mc3	+	-	+	+	-	-
**2**	2mc1	+	-	+	+	-	-
	2mc3	+	-	+	+	-	-
	2mc4	+	-	+	+	+	+
**3**	3mc1	+	-	+	-	+	+
	3mc3	+	-	+	+	-	-
	3mc4.1	+	-	+	-	+	+
	3mc4.2	+	-	+	+	-	-
**4**	4mc1	+	-	+	+	-	-
	4mc2	+	-	+	+	-	-
**5**	5mc1	+	-	-	+	-	-
	5mc2	+	-	-	+	-	-
**6**	6mc1	+	-	-	+	-	-
	6mc4	+	-	-	+	-	-
**7**	7mc1	+	-	+	+	-	-
	7mc2	+	-	+	+	-	-
**8**	8mc1	+	-	+	+	-	-
	8mc4	+	-	+	+	-	-
**9**	9mc1	+	-	+	+	-	-
	9mc3	+	-	+	+	-	-
**10**	10mc1	+	-	+	-	+	-
	10mc2	+	-	+	+	-	-
	10mc3	+	-	+	+	-	-

Sample (Nº)	Isolate ID	IMViC Test					
		Glucose	Acetoin	Mobility	Indole	H_2S	Citrate
**11**	11mc1	+	-	+	+	-	-
	11mc2	+	-	+	+	-	-
**12**	12mc1	+	-	+	-	+	-
	12mc2	+	-	+	NP	+	+
	12mc3	+	-	+	+	-	-
	12mc4	+	-	+	-	+	+
**13**	NA*	NA	NA	NA	NA	NA	NA
**14**	14mc1.1	+	-	+	+	-	-
	14mc3	+	-	+	+	-	-
**15**	15mc1	+	-	+	-	-	-
	15mc2	+	-	+	-	-	-
	15mc3	+	-	+	+	-	-
	15mc5	+	-	+	-	-	-
**16**	16mc1	+	-	+	+	-	-
	16mc3	+	-	+	+	-	-
**17**	17mc1	+	-	+	+	-	-
	17mc2	+	-	+	+	-	-
**18**	18mc1	+	-	-	+	-	-
	18mc3	+	-	+	-	-	-
	18mc4	+	-	+	+	-	-
**19**	19mc1	+	-	+	+	-	-
	19mc2	+	-	-	+	-	-
**20**	20mc1	+	-	+	+	-	-
	20mc2	+	-	+	+	-	-
**21**	21mc1	+	-	+	+	-	-
	21mc2	+	-	+	+	-	-

Sample (Nº)	Isolate ID	IMViC Test					
		Glucose	Acetoin	Mobility	Indole	H_2S	Citrate
**22**	22mc1	+	-	+	+	-	-
	22mc2	+	-	+	+	-	-
	22mc3	+	-	+	-	+	-
	22mc4	+	-	+	+	-	-
**23**	23mc1	+	-	+	+	-	-
	23mc2	+	-	+	+	-	-
**24**	24mc1	+	-	+	+	-	-
	24mc3	+	-	+	+	-	-
**25**	25mc1	+	-	+	+	-	-
	25mc3	+	-	+	+	-	-
**26**	26mc1	+	-	+	+	-	-
	26mc2	+	-	+	+	-	-
**27**	27mc1	+	-	+	+	-	-
	27mc4	+	-	+	+	-	-
**28**	28mc2	+	-	+	+	-	-
	28mc4	+	-	+	+	-	-
**29**	29mc1	+	-	+	+	-	-
	29mc2	+	-	+	+	+	-
	29mc3	+	-	+	+	-	-
	29mc4	+	-	+	+	-	-
**30**	NA[a]	NA	NA	NA	NA	NA	NA
**31**	31mc1	+	-	+	+	-	-
	31mc2	+	-	+	-	-	-
	31mc3	+	-	+	+	-	+
	31mc4	+	-	+	+	-	-
**32**	NA[a]	NA	NA	NA	NA	NA	NA

Sample (Nº)	Isolate ID	IMViC Test					
		Glucose	Acetoin	Mobility	Indole	H_2S	Citrate
**33**	33mc1	+	-	+	+	-	-
	33mc2	+	-	+	-	+	+
	33mc3	+	-	+	+	-	+
	33mc4	+	-	+	+	-	-
**34**	34mc1	+	-	+	+	-	-
	34mc3	+	-	+	+	-	-
**35**	35mc1	+	-	+	+	-	-
	35mc3.1	+	-	+	+	-	-
**36**	36mc1	+	-	+	+	-	-
	36mc3	+	-	+	+	-	-
**37**	37mc1	+	-	+	+	-	-
	37mc2	+	-	+	+	-	-
**38**	38mc1	+	-	+	+	-	-
	38mc2	+	-	-	+	-	+
	38mc3	+	-	+	-	+	+
	38mc4	+	-	-	+	-	-
**39**	39mc1	+	-	+	+	-	-
	39mc2	+	-	+	-	+	-
	39mc3	+	-	+	+	-	-
	39mc4	+	-	-	-	-	+
**40**	40mc1	+	-	+	+	-	-
	40mc2	+	-	+	-	+	-
	40mc3	+	-	+	+	-	-
	40mc4	+	-	+	+	-	-
**41**	41mc1	+	-	+	+	-	-
	41mc4	+	-	+	+	-	-

**42**	42mc1.1	+	-	+	+	-	-
	42mc2	+	-	+	-	+	+
	42mc3	+	-	+	-	+	+
	42mc4	+	-	+	+	+	-
**43**	43mc1	+	-	+	+	+	-
	43mc2	+	-	+	-	-	-
	43mc3.1	+	-	+	-	-	-
	43mc4	+	-	+	-	-	-

Legend: Positive (+); Negative (-); Not Performed (NP); Not Applicable (NA); No bacterial growth on MacConkey agar (*); no lactose-fermenting colonies detected on MacConkey agar (a)
