# Supplementary material for: Bubulcus ibis, Ciconia ciconia and Erinaceus europaeus from a Wildlife Recovery Center in Portugal as Potential Carriers of Resistant Escherichia coli
Source: Vet Sci. 2025 Aug 23;12(9):799. doi: 10.3390/vetsci12090799 (PMC12474354; doi:10.3390/vetsci12090799)
Supplement: Supplementary file 1 [file vetsci-12-00799-s001.zip › Supplementary File S3.pdf]

**Supplementary file S3.** Phenotypic resistance and virulence profiles of 75 selected *E. coli* isolates obtained from

| Sample (Nº) | Isolate ID | MAR Index | MDR isolate | Resistance Profile | | V. Index | Virulence Profile | | | | | |
|---|---|---|---|---|---|---|---|---|---|---|---|---|
| | | | | R | I | | GEL | DNAse | BF | PT | HEM | LEC |
| **1** | 1mc1 | 0,17 | - | AMC-AMP | - | 0 | - | - | - | - | - | - |
| | 1mc3 | 0,42 | MDR | AMP-STX-TE-C | AMC | 0 | - | - | - | - | - | - |
| **2** | 2mc1 | 0,17 | - | AMC-AMP | - | 0 | - | - | - | - | - | - |
| | 2mc3 | 0,08 | - | AMP | - | 0 | - | - | - | - | - | - |
| **3** | 3mc3 | 0,08 | - | AMP | - | 0 | - | - | - | - | - | - |
| | 3mc4.2 | 0,08 | - | - | AMP | 0 | - | - | - | - | - | - |
| **4** | 4mc1 | 0,08 | - | AMP | - | 0 | - | - | - | - | - | - |
| | 4mc2 | 0,08 | - | - | AMP | 0,17 | - | - | - | + | - | - |
| **5** | 5mc1 | 0,17 | - | AMP | AMC | 0,17 | - | - | + | - | - | - |
| | 5mc2 | 0,08 | - | - | AMP | 0 | - | - | - | - | - | - |
| **6** | 6mc1 | 0,17 | - | TE-C | - | 0 | - | - | - | - | - | - |
| | 6mc4 | 0,17 | - | TE-C | - | 0 | - | - | - | - | - | - |
| **7** | 7mc1 | 0 | - | - | - | 0 | - | - | - | - | - | - |
| | 7mc2 | 0 | - | - | - | 0,17 | - | - | - | + | - | - |
| **8** | 8mc1 | 0,17 | - | AMP | AMC | 0 | - | - | - | - | - | - |
| | 8mc4 | 0,08 | - | AMP | - | 0 | - | - | - | - | - | - |
| **9** | 9mc1 | 0,08 | - | - | AMP | 0 | - | - | - | - | - | - |
| | 9mc3 | 0 | - | - | - | 0 | - | - | - | - | - | - |
| **10** | 10mc2 | 0,08 | - | - | AMP | 0 | - | - | - | - | - | - |
| | 10mc3 | 0 | - | - | - | 0 | - | - | - | - | - | - |
| **11** | 11mc1 | 0,08 | - | AMP | - | 0 | - | - | - | - | - | - |
| | 11mc2 | 0,08 | - | AMP | - | 0 | - | - | - | - | - | - |
| **12** | 12mc3 | 0 | - | - | - | 0,33 | - | - | + | + | - | - |
| **14** | 14mc1.1 | 0,08 | - | AMP | - | 0 | - | - | - | - | - | - |
| | 14mc3 | 0,08 | - | - | AMP | 0,17 | - | - | - | + | - | - |

| Sample (Nº) | Isolate ID | MAR Index | MDR isolate | Resistance Profile | | V. Index | Virulence Profile | | | | | |
|---|---|---|---|---|---|---|---|---|---|---|---|---|
| | | | | R | I | | GEL | DNAse | BF | PT | HEM | LEC |
| **15** | 15mc3 | 0,08 | - | - | AMP | 0 | - | - | - | - | - | - |
| **16** | 16mc1 | 0,50 | MDR | AMP-STX-ENR-MAR-CN-C | - | 0,17 | - | - | + | - | - | - |
| | 16mc3 | 0 | - | - | - | 0 | - | - | - | - | - | - |
| **17** | 17mc1 | 0 | - | - | - | 0 | - | - | - | - | - | - |
| | 17mc2 | 0 | - | - | - | 0,17 | - | - | - | - | β | - |
| **18** | 18mc1 | 0,08 | - | AMP | - | 0 | - | - | - | - | - | - |
| | 18mc4 | 0 | - | - | - | 0 | - | - | - | - | - | - |
| **19** | 19mc1 | 0 | - | - | - | 0 | - | - | - | - | - | - |
| | 19mc2 | 0 | - | - | - | 0 | - | - | - | - | - | - |
| **20** | 20mc1 | 0,42 | MDR | AMP-STX-TE | AMC-ENR | 0 | - | - | - | - | - | - |
| | 20mc2 | 0,17 | - | AMP | AMC | 0 | - | - | - | - | - | - |
| **21** | 21mc1 | 0,08 | - | - | AMP | 0,17 | - | - | - | - | β | - |
| | 21mc2 | 0,08 | - | - | AMP | 0 | - | - | - | - | - | - |
| **22** | 22mc1 | 0,08 | - | - | AMP | 0 | - | - | - | - | - | - |
| | 22mc4 | 0 | - | - | - | 0,17 | - | - | + | - | - | - |
| **23** | 23mc1 | 0,42 | MDR | AMP-STX-TE-C | AMC-ENR | 0 | - | - | - | - | - | - |
| | 23mc2 | 0,17 | - | AMP-TE | - | 0 | - | - | - | - | - | - |
| **24** | 24mc1 | 0 | - | - | - | 0 | - | - | - | - | - | - |
| | 24mc3 | 0,08 | - | - | AMP | 0 | - | - | - | - | - | - |
| **25** | 25mc1 | 0,08 | - | TE | - | 0 | - | - | - | - | - | - |
| | 25mc3 | 0 | - | - | - | 0,17 | - | - | - | + | - | - |
| **26** | 26mc1 | 0,08 | - | - | AMP | 0 | - | - | - | - | - | - |
| | 26mc2 | 0,08 | - | AMP | - | 0,17 | - | - | - | - | β | - |
| **27** | 27mc1 | 0,08 | - | - | AMP | 0 | - | - | - | - | - | - |
| | 27mc4 | 0 | - | - | - | 0 | - | - | - | - | - | - |

| Sample (Nº) | Isolate ID | MAR Index | MDR isolate | Resistance Profile | | V. Index | Virulence Profile | | | | | |
|---|---|---|---|---|---|---|---|---|---|---|---|---|
| | | | | R | I | | GEL | DNAse | BF | PT | HEM | LEC |
| **28** | 28mc2 | 0,08 | - | **AMP-STX** | **-** | 0 | - | - | - | - | - | - |
| | 28mc4 | 0,08 | - | **-** | **AMP** | 0 | - | - | - | - | - | - |
| **29** | 29mc1 | 0,08 | - | **C** | **-** | 0,33 | - | - | + | + | - | - |
| | 29mc3 | 0,17 | - | **AMP** | **MAR** | 0 | - | - | - | - | - | - |
| **31** | 31mc1 | 0 | - | **-** | **-** | 0 | - | - | - | - | - | - |
| | 31mc3 | 0 | - | **-** | **-** | 0 | - | - | - | - | - | - |
| **33** | 33mc1 | 0 | - | - | - | 0,17 | - | - | - | + | - | - |
| | 33mc4 | 0 | - | - | - | 0 | - | - | - | - | - | - |
| **34** | 34mc1 | 0,08 | - | AMP | - | 0 | - | - | - | - | - | - |
| | 34mc3 | 0,08 | - | - | AMP | 0 | - | - | - | - | - | - |
| **35** | 35mc1 | 0 | - | - | - | 0 | - | - | - | - | - | - |
| | 35mc3.1 | 0,17 | - | AMC-AMP | - | 0 | - | - | - | - | - | - |
| **36** | 36mc1 | 0,17 | - | AMP | CN | 0 | - | - | - | - | - | - |
| | 36mc3 | 0 | - | - | - | 0 | - | - | - | - | - | - |
| **37** | 37mc1 | 0,08 | - | TE | - | 0,17 | - | - | - | + | - | - |
| | 37mc2 | 0 | - | - | - | 0 | - | - | - | - | - | - |
| **38** | 38mc1 | 0,08 | - | - | AMP | 0 | - | - | - | - | - | - |
| | 38mc4 | 0 | - | - | - | 0 | - | - | - | - | - | - |
| **39** | 39mc1 | 0,08 | - | - | AMP | 0 | - | - | - | - | - | - |
| | 39mc3 | 0 | - | - | - | 0 | - | - | - | - | - | - |
| **40** | 40mc1 | 0 | - | - | - | 0 | - | - | - | - | - | - |
| | 40mc3 | 0 | - | - | - | 0 | - | - | - | - | - | - |
| **41** | 41mc1 | 0,08 | - | AMP | - | 0 | - | - | - | - | - | - |
| | 41mc4 | 0 | - | - | - | 0 | - | - | - | - | - | - |
| **42** | 42mc1 | 0,17 | - | TE | AMC | 0 | - | - | - | - | - | - |

**Legend:** Resistant (R), Intermediate (I), Ampicillin (AMP), Amoxicillin/Clavulanic Acid (AMC), Gentamicin (CN), Tetracycline (TE), Chloramphenicol (C), Enrofloxacin (ENR), Marbofloxacin (MAR), Trimethoprim/Sulfamethoxazole (STX), Positive (+), Negative (−), Gelatinases (GEL), Biofilm (BF), Proteases (PT), Hemolysin (HEM), Lecithinases (LEC), Multiple Antimicrobial Resistance Index (Index MAR), Virulence Index (Index V.), Multidrug-resistant (MDR).
